# Preparation and Evaluation of Azelaic Acid Topical Microemulsion Formulation: In Vitro and In Vivo Study

**DOI:** 10.3390/pharmaceutics13030410

**Published:** 2021-03-19

**Authors:** Wan-Hsuan Hung, Ping-Kang Chen, Chih-Wun Fang, Ying-Chi Lin, Pao-Chu Wu

**Affiliations:** 1 Divison of Pharmacy, Zuoying Branch of Kaohsiung Armed Forces General Hospital, Kaohsiung City 81342, Taiwan; 103534007@gap.kmu.edu.tw (W.-H.H.); u101530009@gap.kmu.edu.tw (C.-W.F.); 2 School of Pharmacy, College of Pharmacy, Kaohsiung Medical University, Kaohsiung City 80708, Taiwan; u107530005@gap.kmu.edu.tw; 3 Doctoral Degree Program in Toxicology, College of Pharmacy, Kaohsiung Medical University, Kaohsiung City 80708, Taiwan; 4 Department of Medical Research, Kaohsiung Medical University Hospital, Kaohsiung City 80708, Taiwan; 5 Drug Development and Value Creation Research Center, Kaohsiung Medical University, Kaohsiung 80708, Taiwan

**Keywords:** O/W microemulsion, topical application, azelaic acid, edema index, stability

## Abstract

The aim of this study was to design oil in water (O/W) microemulsion formulations for the topical administration of azelaic acid. The permeability of azelaic acid through rat skin and the anti-inflammatory activities of the formulations were conducted to examine the efficacy of the designed formulations. Skin irritation and stability tests were also performed. The permeability of azelaic acid was significantly increased by using O/W microemulsions as carriers. The edema index of ear swelling percentage was significantly recovered by the 5% drug-loaded formulation and a 20% commercial product, demonstrating that the experimental formulation possessed comparable effect with the commercial product on the improvement of inflammation. The experimental formulation did not cause significant skin irritation compared to the negative control group. Moreover, the drug-loaded formulation also showed thermodynamic stability and chemical stability after storage for 30 days. In conclusion, the O/W microemulsion was a potential drug delivery carrier for azelaic acid topical application.

## 1. Introduction

Azelaic acid (C_9_H_16_O_4_, MW 188.22 g/mole, pKa values of 4.53 and 5.33) is a straight-chained dicarboxylic acid. Azelaic acid possesses anti-inflammatory properties, by its activity as a free oxygen radical scavenger, antibacterial effects against *Staphylococcus epidermidis* and *Propionibacterium acnes*, and keratolytic activity [1,2]. Azelaic acid was thereby topically used for the treatment of acne and rosacea. A 15% Azelaic acid gel was first approved on December 24, 2002 for the treatment of rosacea [3]. Since then, a 20% cream has also been approved for the treatment of acne. Due to its low-water solubility, about 0.24 g/100 mL at 25 °C, and limited penetration across the stratum corneum (SC), many formulations have been proposed, including microemulsion, liposome, gel, ethosome, liquid crystal and foam formulations [4,5,6,7,8], to increase the solubility and permeability of azelaic acid through the skin and to reduce the amount of drug used in the formulations.

In the pharmaceutical industry, the choice of an appropriate dosage form not only depends on the therapeutic effect but also many other factors, including the manufacturing process, cost, and patient attractiveness and compliance. Therefore, choosing appropriate excipients and ingredients is a crucial step in the design of appropriate formulations. Microemulsion is a promising nanocarrier for the topical application of insoluble therapeutic compounds [9,10,11,12,13]. The composition is simple, consisting of only the oil phase, surfactant, cosurfactant and water phase, and can be easily prepared via mild stirring. Oil in water (O/W) microemulsions can be spontaneously formed by “solubilizing” oil molecules with a mixture of surfactants, cosurfactants, and cosolvents [14,15]. Since the continuous phase is water, the formulation is less oily and easy to be removed from the applied sites. Hence, O/W microemulsions were examined as a drug carrier in this study to simultaneously enhance solubilization and skin permeability of azelaic acid.

Isopropyl myristate is a polar emollient and potent skin enhancer that is used in cosmetic and topical medicinal preparations where good absorption into the skin is desired [16]. Cremophor^®^ EL is ethyl oleate and polyoxyethylene castor oil with strong emulsifying properties that is commonly used as an excipient for the creation of microemulsions [16]. Transcutol^®^ is a highly purified and safe diethylene glycol monoethyl ether that can be used with concentrations up to 49.9% for topical administration in the Inactive Ingredients Database of the US Food and Drug Administration (FDA). Transcutol^®^ possesses good solvent properties for poorly water-soluble compounds and can promote drug permeation and produce a drug depot effect [17]. Previous research had reported microemulsions containing isopropyl myristate, cremophorEL or trancutol that possessed significantly enhancement in permeability [18,19]. Hence, the isopropyl myristate /cremophorEL/trancutol microemulsions were used as drug carrier to improve the solubility and permeability of azelaic acid in this study. In this manuscript, we performed in vitro permeation, in vivo anti-inflammatory test, as well as skin irritation and stability tests to evaluate the efficacy of the designed microemulsion formulation.

## 2. Materials and Methods

### 2.1. Materials

Azelaic acid was purchased from Acros Organic (Carlsbad, CA, USA). Isopropyl myristate was obtained from Tokyo Chemical Industry (Tokyo, Japan). Cremophor EL was purchased from Fluka (Forest Parkway College Park, GA, USA). Transcutol HP was from Gattefosse (Chemin de Genas, Saint Priest, France). Polyethylene glycol 400 was from Showa (Osaka, Japan). The commercial cream product (Skinoren^®^) containing 20% azelaic acid was obtained from Bayer Co., Ltd. (Taiwan). All other chemicals and solvents were of analytical reagent grade.

### 2.2. Preparation of Azelaic Acid-Loaded Microemulsions

Azelaic acid-loaded formulations were prepared by a spontaneous emulsion method. The oil phase (isopropyl myristate), surfactants and cosurfactant were mixed well by vortexing at room temperature. Distilled water was then slowly added to the mixture and vortexed for 1–2 min to get a homogeneous mixture: blank microemulsion. Azelaic acid of 0.5 g was dissolved in the blank microemulsion of 9.5 mL by shaking for 12 h. There was no precipitate observed in the final drug-loaded formulations. The compositions of the azelaic acid-loaded microemulsions are listed in Table 1.

### 2.3. Mean Droplet Size Determination

Mean droplet size distribution and polydispersity index (PDI) of the azelaic acid-loaded formulations were measured by Malvern Zetasizer 3000HSA equipped with laser diffraction (Malvern Panalytical, Malvern, UK). Three milliliters of test samples were loaded into a cuvette and placed in the scattering chamber. The intensity of the light scattering from the helium-neon laser were set at an angle of 90° and a wavelength of 633 nm.

### 2.4. Determination of Viscosity

Viscosities of azelaic acid-loaded formulations were determined using cone-plate of viscometer (Model LVDV-II, Brookfield Ametek, USA). Tested samples were placed into the cone-plate and heated to 37 °C, by a thermostatic pump for three mins. Readings were recorded 30 s after the measurement reached 20 rpm.

### 2.5. In Vitro Skin Permeation Study

All experiments in this study were approved on 1 October 2019 (No. 108075) by the Institutional Animal Care and Use Committee of Kaohsiung Medical University (Kaohsiung, Taiwan). The committee confirmed that all experiments followed the guidelines as set forth by the Guide for Laboratory Fact lines and Care. For in vitro skin permeation experiment, the modified transdermal Franz diffusion cell was used to determine the permeability of azelaic acid-loaded formulations: the receptor cell contained 20 mL of pH 7.4 phosphate buffer with 30% ethanol. The effective diffusion area of the cell was 3.46 cm^2^_,_ and the drug solution also contained 30% ethanol. The abdominal skin excised from Sprague Dawley rats (275–300 g) was used as the barrier membrane. One mL of the test sample was placed in the donor cell, which was then occluded by parafilm. The receptor cell was stirred at 550 rpm by a magnetic stirrer and maintained at 37 ± 0.5 °C, by circulating water with a thermostatic pump. At predetermined intervals, i.e., 1, 2, 3, 4, 5, 6, 8, 12, and 24 h, 1 mL of the receptor medium was withdrawn and replaced with the same volume of fresh medium via the sampling port. The drug content was analyzed by a HPLC method modified from a previous study [20]. All experiments were repeated in triplicate.

### 2.6. Condition of HPLC Analysis of Azelaic Acid

A Hitachi HPLC system equipped with a pump (L-2130), autosampler (L2200) and UV detector (L7420) were used for azelaic acid analysis. Twenty 20 µL of each sample was injected. The mobile phase comprised of pH 3.0 phosphoric acid solution and acetonitrile (70:30), which was eluted at a flow rate of 0.8 mL/min through a stationary phase of LiChroCART^®^ RP-18e column (4′250 mm I.D. wih particle size of 5 μm) at ambient temperature i.e., 25 °C, Analysis was conducted at a detection wavelength of 215 nm. Benzoic acid of 50 µg/mL was used as internal standard. The limit of quantitation was 10 µg/mL. The linearity was r^2^ = 0.9992 ranged from 100 to 1000 µg/mL of azelaic acid. The precision of coefficient of variation and accuracy of relative error were less than 3.67% and 4.22%, respectively.

### 2.7. Pharmacodynamic Evaluation

Twenty-four Balb/c mice were randomized into four groups for a single dose of test formulations (Formulation blank, Formulation containing 5% drug, Commercial product, and untreated). A 10 μL of 0.8% croton oil (inflammatory inducer) in acetone was painted onto the inner surface of the left ear of each mouse to induce rosacea disease model. Fifteen mins after the induction, 50 μL of the different formulations (equal to 2.5 mg drug) were topically applied to the left ear. At 0, 4, 8 and 24 h, the thickness of each ear, near the top of the ear distal to the cartilaginous ridges, was measured. The change (swelling %) in ear thickness of the left ear from the right ear was taken as an edema index [21,22].

Swelling % = (right ear thickness − left ear thickness)/right ear thickness × 100%

### 2.8. Skin Irritation Determination

Skin irritation caused by the tested formulations was evaluated by histological microscopy. The Sprague-Dawley (SD) rats were divided into four groups: positive control group (treated with 0.8% paraformaldehyde), negative control groups (water-treated group), and the tested groups (treated with drug-free and drug-loaded microemulsions, and a commercial product) [23,24]. For testing, 0.5 mL of the formulations was evenly spread on the shaven abdomen skin of 2.54 cm^2^ and then occluded by parafilm for 24 h [24,25]. The mice were then sacrificed, and the applied skin tissue was excised for histological examination with the skin tissue being fixed in 10% buffered formaldehyde solution for at least 24 h before routine processing. Tissue samples were fixed, rinsed with running distilled water, dehydrated using with a graded series of ethanol solution and embedded in paraffin. The tissue samples were cut into sections of 20 μm, rehydrated, and stained with hematoxylin and eosin for histological evaluation. All samples were examined by light microscope (Nikon Eclipse Ci, Tokyo, Japan).

### 2.9. Stability of Azelaic Acid-Loaded Formulation Determination

To quickly evaluate the stability of the M3 microemulsion, thermodynamic stability tests including centrifugation and freeze-thaw cycle methods were performed [26,27]. Briefly, the selected drug-loaded formulation was centrifuged at 3,500 rpm for 30 min and three cycles conducted between freeze temperature (−21 °C) and room temperature 25 °C with storage at each temperature for no less than 24 h. The phase separation, transparency, and precipitation of drug from formulations were investigated. For the chemical stability test, azelaic acid-loaded microemulsion was stored at 25 ± 1 °C for 30 days. The concentration of azelaic acid in the microemulsion was determined by HPLC.

### 2.10. Data Analysis

All data were represented as mean values ± standard deviation. The ANOVA test and post hoc turkey test provided by SPSS-Statistic software 22.0 (IBM Corp, Armonk, NY, USA) was used for analysis of the differences among the tested formulations. The probability of 0.05 (*p* < 0.05) was considered as the significance level.

## 3. Results and Discussion

### 3.1. Physicohemical Characterization

The composition, droplet size and PDI are presented in Table 1. The PDI values of all formulations were smaller than 0.3, indicating that the mixtures achieved homogenization. Increasing the concentration of ethanol increased the droplet size. The droplet size of all formulations, except for M4 (PG replaced PEG as the cosurfactant) were in submicro-scale. The phenomenon may be attributed to the higher viscosity of PG increased the viscosity of the microemulsion system and gives the higher possibility of the gel formation with an increased droplet size [28,29].

### 3.2. In Vitro Permeation Study

**Figure 1:**
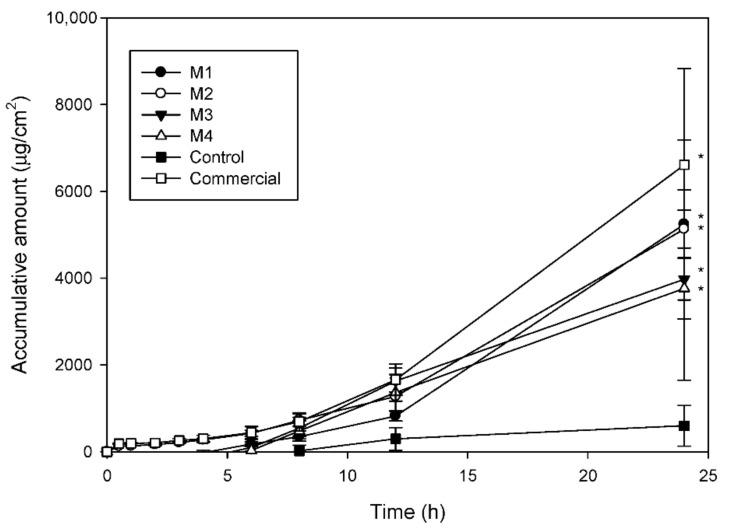
In vitro penetration-time profile of 5% azelaic acid-loaded formulations and control solution containing 5% drug and 30% ethanol through rat skin.* significant difference when compared with control group.

The in vitro penetration-time profiles of azelaic acid-loaded formulations and the control group, drug solution containing 30% ethanol, through rat skin are presented in Figure 1. For the control group, the lag time (first time the drug is detected) was about 8 h and the cumulative amount at 24 h was about 598.9 μg/mL/cm^2^, showing the difficulty of azelaic acid to transport through rat skin. When using microemulsions as carrier, lag time was shortened to 2~6 h and the cumulative amounts of azelaic acid were significantly increased. The results were consistent with previous reports indicating that microemulsions can improve the permeability of therapeutic compounds. The combined effect of hydrophilic and lipophilic components of the microemulsions may act as penetration enhancers. In addition, microemulsions with submicron scale-size droplet can reduce the contact angle between skin and formulation, resulting in faster permeation [30,31].

To compare the effect of excipients, it was found that when PG was used instead of PEG 400, the two penetration curves were similar but the particle size increased, and the appearance became opaque. By incorporating 5–10% ethanol in the drug-loaded microemulsions, it was found that the cumulative amount decreased as ethanol level increased. Previous studies reported that when the excess amount of cosurfactant was added into microemulsion; the thermodynamic activity of the drug in microemulsion may be decreased and resulted in decreased penetration rate [13,32]. In compared with the commercial product, the cumulative amount at 24 h of M3 was closed to commercial product. Therefore, the formulation was selected for further evaluation.

### 3.3. Pharmacodynamic Evaluation

**Figure 2:**
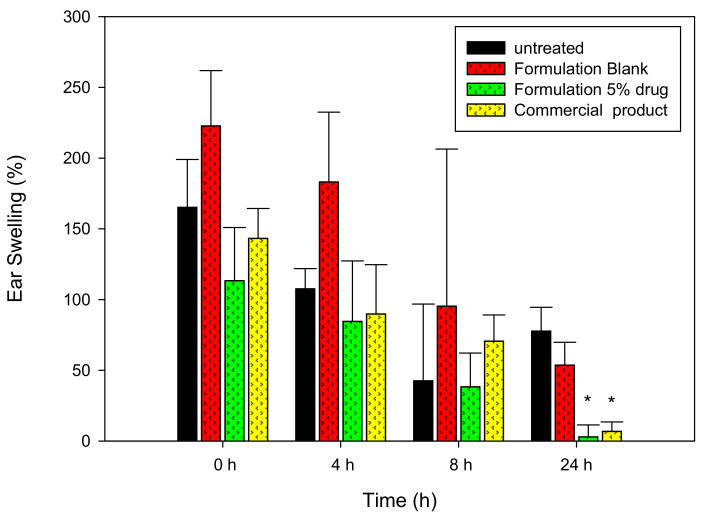
The ear-swelling (%) of mice treated with different formulations. * Significant difference shown when compared with untreated samples (*p* < 0.05).

In this study, the cutaneous polymorphonuclear leukocyte inflammation caused by croton oil was used to evaluate effectiveness of experimental formulations [21], with the ear-swelling data taken as an inflammatory index. As shown in Figure 2, after treatment with croton oil, inflammation was obvious. Without treatment, inflammation although decrease over time but still existed after 24 h. The application of blank M3 did not improve the inflammation condition compared to the control group. On the contrary, 5% of azelaic acid-loaded formulation and the commercial product exhibited a significant reduction (*p* < 0.05) on the swelling after 24 h of application. The results demonstrated that the 5% experimental preparation had comparable effects to the commercial cream containing 20% azelaic acid. The nanocarrier of the microemulsion could improve the drug permeability through the skin.

### 3.4. Skin Irritation

**Figure 3:**
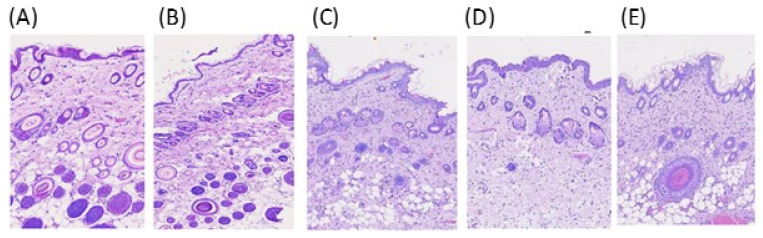
The photomicrographs of rat skin section with different treatments viewed under a light microscope (4 × 10): (**A**) the intact skin showing integrity; (**B**) the paraformaldehyde-treated skin showing slight edematous exfoliation of the stratum corneum (SC) and loosely textured collagen in the dermis; (**C**) blank M3-treated skin showing well-defined epidermal and dermal layers; (**D**) M3 containing 5% drugtreated; (**E**) commercial formulation-treated, showed slight edematous exfoliation of the SC.

Irritation is an important issue for topically applied formulations. Histological examination of skin after 24 h application was conducted in this study to assess the potential irritating effects of the designed formulations. The skin samples treated with distilled water and 0.8% paraformaldehyde aqueous solution were used as negative control and positive control, respectively [25,32]. The histopathological image of the treated rat skin sections are represented in Figure 3A,E. In the negative control, well-defined epidermal and dermal layers were observed (Figure 3A), while the positive control showed mild edematous exfoliation in the SC layer and loose collagen texture in the dermis layer (Figure 3B). For the commercial product (Figure 3E), a slight edematous exfoliation of the SC was found. Non-significant edema and erythema were found in the blank microemulsion of M3- and M3 formulation -treated skin sections (Figure 3C,D) when compared to the distilled water-treated group of Figure 3A. The observations indicated that the designed microemulsion formulation appeared to be a safe carrier for the topical use of azelaic acid.

### 3.5. Stability of the Azelaic Acid-Loaded Formulations

After centrifugation at 3500 rpm for 30 min and three freeze-thaw cycles [26,27], the physical appearances of formulations were unchanged in terms of phase separation and transparency, demonstrating that the azelaic acid-loaded microemulsion possessed thermodynamic stability; moreover, drug precipitation was not observed. After storage at 25 °C for 30 days, the azelaic acid content was 96.3 ± 4.16% and the droplet size and viscosity of the formulation did not change significantly, indicating that the tested azelaic acid-loaded formulations were considered stable.

## 4. Conclusions

The cumulative amount of the control group (azelaic acid dissolved in 30% ethanol) through rat skin at 24 h was low at the in vitro permeation study, indicating the difficulty of azelaic acid to transport across the skin barrier. With the microemulsion carrier, the permeability of azelaic acid was remarkably improved. The composition of the microemulsion formulations affected the permeation capacity of the drug. Through an in vivo anti-inflammatory test in which ear swelling (%) was used as an edematous index, the drug-loaded formulation significantly reduced ear swelling (%) compared to the untreated group 24 h after the application. The drug-loaded microemulsion formulation did not induce significant skin irritation and exhibited considerable stability. From the above results, the O/W microemulsion could be a promising drug delivery carrier for topical application of azelaic acid.

## Figures and Tables

**Table 1:** The composition, cumulative amount and mean droplet size of azelaic acid-loaded formulations.

Formulation	M1	M2	M3	M4
IPM	10	10	10	10
Cremophor EL	20	20	20	20
Transcutol	10	10	10	10
PEG	26	26	26	-
PG	-	-	-	26
Ethanol	-	5	10	10
Water	34	29	24	24
Azelaic	5	5	5	5
Q_24h_(μg/cm^2^)	5239.5 ± 3590.9	5131.1 ± 435.4	3974.9 ± 479.8	3764.8 ± 707.8
Droplet size (nm)	220.7 ± 4.6	243.2 ± 1.3	700.7 ± 13.6	8674.6 ± 475.9
PDI	0.27 ± 0.01	0.28 ± 0.00	0.24 ± 0.02	3.28 ± 0.31
Viscosity (cps)	247.4 ± 24.1	187.5 ± 10.7	145.4 ± 5.0	-

IPM: isopropyl myristate; Q_24h_: drug cumulative amount at 24 h; PDI: polydispersity index.

## Data Availability

Not applicable.
